# Correction of Midline Deviation and Unilateral Crossbite Treated with Fixed Appliance

**DOI:** 10.1155/2023/5620345

**Published:** 2023-02-18

**Authors:** Domenico Ciavarella, Marta Maci, Laura Guida, Angela Pia Cazzolla, Eleonora Lo Muzio, Michele Tepedino

**Affiliations:** ^1^Department of Clinical and Experimental Medicine, Dental School of Foggia, University of Foggia, Foggia, Italy; ^2^Department of Translational Medicine and for Romagna, School of Orthodontics, University of Ferrara, Ferrara, Italy; ^3^Department of Biotechnological and Applied Clinical Sciences, University of L'Aquila, L'Aquila, Italy

## Abstract

The following case report describes the treatment of a 20-year-old man with dental and facial asymmetry. Patient presented upper dental midline shifted 3 mm to right side and the lower one 1 mm to left side, skeletal class I, molar class I and canine class III on the right side, molar class I and canine class II on the left side, and upper and lower crowding on teeth #12, #15, #22, #24, #34, and #35 in crossbite. The treatment plan provided four extractions: in the superior arch the right second and the left first premolar and in the lower arch first premolars on the left and right sides. Wire-fixed orthodontic device was used in combinations with coils to correct the midline deviation and to close post-extractive spaces, avoiding miniscrew implants. At the end of the treatment, optimal functional and aesthetic results were obtained: realignment of the midline improved facial symmetry, correction of the crossbite on both sides, and a good occlusal relationship.

## 1. Introduction

Midline deviations is one of the more difficult, common, and persistent problem that all orthodontists must face, and it might be seen in all types of malocclusions [1]. It is very often seen lack of midline correspondence between the upper and lower arches or even if both corresponding may not be symmetrically placed in the face [2].

Dental asymmetry could be caused by unilateral crossbites, arch form not symmetric or upper and lower arches not congruent. In order to correct midline deviation, the first step in the diagnosis and treatment planning of all patients should be to make correct differential diagnosis to identify these asymmetries, to differentiate between those that are of a dental or a skeletal origin, and to evaluate the effects on the occlusion [1, 2].

It is only then that the clinician can make a valid decision concerning the need for surgery or a nonsurgical approach: if the choice of treatment is non-surgical, the clinician has to decide if the extraction is the treatment of choice.

The causes of these asymmetries can be multiple:

1. Skeletal origin: asymmetric maxilla or, more likely, an asymmetric mandible [2].
2. Lateral mandibular deviation [1].
3. Asymmetries of the upper and/or lower arch [1].
4. Abnormal dental eruption, premature loss of primary teeth, or loss of permanent teeth [2].
5. Tipping and/or drifting on the upper and/or lower incisor [1].
6. Any combination of these factors [1].

## 2. Case Presentation

### 2.1. Diagnosis and Etiology

A 20-year-old male patient presented to our clinic with chief complaints of mandibular deviation and crowding. On extraoral examination, facial asymmetry was observed, and his chin was deviated to the left side (Figure 1(a)). The patient was evaluated by a thoughtful clinical evaluation: patient had not temporomandibular joint (TMJ) problems or pains or sounds during protrusion and lateral movements. Class I molar and class III canine on the right side, class I molar and canine class II on the left side were seen. Additionally, there was a lower midline shift 1 mm to left side and upper midline shift 3 mm to right side. A crossbites of teeth #12, #15, #22, #24, #34, and #35 were present (Figures 1(b) and 1(c)). Class III relationship in the right side is associated to an asymmetric growth of the jaw, to upper arch contraction, and to midline maxillomandibular deviation.

The intraoral examination confirmed on the right side class I molar and class III canine, on the left side class I molar and class II canine, teeth #12, #15, #22, #24, #34, and #35, in crossbite (Figures 1(b) and 1(c)).

Cephalometric measurements (e.g., latero-lateral cephalometric analysis, see Table 1) revealed a skeletal class I malocclusion (ANB 2.5°), with a biretrusion: (SNA 76.1° and SNB 73.6°), and a hyperdivergent growth pattern (S-NA and GO-ME 40.3°). The Wits appraisal was 3.6. The distance between the upper incisors and NA-PG is 8.77 mm, and lower incisor was 4.74 mm. The UI^SN was 100.45° and LI^GOME was 91.7°. The interincisal angle was 127.57°. Posterior–anterior cephalometry (Table 2) showed good maxillo-mandibular widths (maxillary qidth = 66.1 and mandibular width = 93.1) occlusal plane canting (occlusal plane tilt = 4.6°), and mandibular lateral deviation and slight differences in the distances of the molars.

The panoramic radiograph showed the presence of all permanent teeth, except teeth #18 and #48 (Figure 2).

After radiological and facial evaluation, the patient was diagnosed with a class I malocclusion, presenting a dolichofacial, asymmetrical, and concave profile with bi-retrusion. The etiology of the asymmetry is multifactorial linked to a combination of skeletal and dental factors.

### 2.2. Treatment Objectives

The treatment objectives were:

- to correct upper and lower midline deviation;
- to align the upper and lower crowding;
- to correct the crossbite;
- improvement of the profile and aesthetic; and
- good functional occlusion.

To achieve these objectives it has been proceeded with extractions of teeth #15 and #24 in the upper arch and of teeth #34 and #44 in the lower arch, subsequent symmetrization of the arches and closure of the spaces. The extractions were done to solve severe crowding. In the maxillary arch, asymmetric extractions were done to correct the midline deviation; in the lower arch, symmetric extractions of bicuspid were done to reduce the class III tendency of the patient.

### 2.3. Therapeutic Alternatives

The therapeutic alternatives were:

- Orthodontic treatment with four asymmetric extractions and closing spaces with temporary anchorage devices (TADs).
- Orthodontic treatment without extractions treatment using no friction brackets appliance in upper arch even if the midline correction would be difficult.
- Orthodontic treatment without extractions using in upper arch, TADs on the left side to obtain asymmetric distalization; and in lower arch, interproximal reduction) and no friction brackets appliance.
- Four asymmetric extractions and orthognathic surgery.

Whenever the facial asymmetry is severe, it is better to solve the problem through orthodontics decompensation and asymmetric jaw surgery [3]. In the other side, if the asymmetry is mild, it may be difficult for the patient to agree the surgery, and occlusal problems may be corrected through orthodontic treatment alone: severe dental midline deviation relative to the face very often require teeth extractions. Small asymmetries can be corrected with intermaxillary elastics or mini-implants, asymmetric extractions, and stripping [4]. It is important to maintain the proper anchorage control, during the necessary asymmetrical movement, to achieve an ideal class first occlusion. In the most of cases, it is difficult to improve facial asymmetry without orthognathic surgery, but in some patients, it can be obtained by orthodontic treatment alone [5].

### 2.4. Treatment Progress

Fixed orthodontic treatment started with fixed appliance in the upper and inferior arches. Four premolars (teeth #15, #24, #34, and #44) were extracted to correct the inferior and superior midline and to reduce crowding. Furthermore, in an initial phase, a lingual arch was used as an anchorage. After extractions, the arches were symmetrized with coils and then space closures were done.

The wire sequence adopted for alignment and leveling was the following:

- 0.014 NiTi wire upper and lower arch.
- 0.016 NiTi wire upper and lower arch.
- 0.016 × 0.016 stainless steel wire upper and lower arch with asymmetric lace backs and then asymmetric elastics.
- 0.020 × 0.020 NiTi wire upper and lower arch with band back, coil spring open between teeth #21 and #23 to correct the midline, and lacebacks between teeth #47 and #43 to distalize the canine was performed (Figure 3).
- Coil spring open between teeth #12 and #13 to correct the midline. In the lower arch coil, spring closed between teeth #47 and #43 and hook to close post-extractive space (Figure 4).
- 19 × 25 stainless steel posted in lower arch with coils to close the post-extractive space between teeth #47 and #43.
- An antispee wire 19 × 25 stainless steel wire in upper arch and antispee wire 16 × 22 stainless steel in lower arch.
- 0.015 NiTi wire upper arch.
- 0.016 × 0.022 NiTi wire in lower arch with antispee.
- 0.019 × 0.025 upper arch stainless steel wire with superspee and surgery arch lock in distal position to teeth #22 to close the space with a traction module “coil spring” 200 g with 0.012 × 0.30 section: medium strength.

At the end of the treatment, 36 months long, Hawley retainers were delivered to the patient.

## 3. Results

At the end of treatment, a good occlusal relationship was achieved, with the correction of the crowding, and of occlusal plane canting, coincidence of midlines, and the correction of canine class III and II malocclusion, without the need of miniscrews. The occlusion was stable and functional and the facial aesthetic was optimal (Figures 5(a), 5(b), 5(c), and 6).

Final cephalometric analysis showed values for ANB 2.1°; SNA 76.7°; SNB 74.6°; and a hyperdivergent growth pattern (S-NA and GO-ME 40.6°). The wits appraisal was 2.9.

The interincisal angle was 125.09° (Table 1). The UI^SN was 104.67° and LI^GOME was 89.62°. The patient compliance was excellent. Final postero-anterior cephalometric analysis showed an improvement of skeletal relationships both in the sagittal and transversal planes and correction of occlusal plane canting.The 5-year follow up showed the stability of the results obtained (Figure 7).

## 4. Discussion

Correction of dental asymmetries is considered a difficult process, due to misdiagnosis and poorly planned treatment mechanics [6].

Patients with dentoalveolar asymmetries quite often present some of the most biomechanically challenging situations, which may require different therapeutic options and include combined orthodontic-surgical treatments. Where the patient refuses orthodontic-surgical treatment, teeth extractions could be fully correct malocclusions as in the case-report presented [7].

In order to correct the midline deviation and to solve anterior crowding, teeth extraction was necessary: in the superior arch the second right, due to the position plus crowding and the first left premolar were extracted, whereas in the lower arch, the first left and right premolars were extracted. The asymmetric extractions allowed to correct midline deviation and anterior crowding favoring unilateral movement of the posterior teeth without using intermaxillary elastics [1], obtain occlusal stability and a good response on patient profile [8–10].

Extraction of the first four premolars could have been an another option because it offered the advantage of using a simpler mechanism than control of anchorage and to preserve the contact point between the second premolar and the first molar (more anatomically correct and preferable than the contact of the first premolar with the first molar) [4, 11, 12]. Usually, the first premolars are chosen because of their position and compatible size with most types of cases. Although as a rule, the extraction of second premolars is not indicated for cases with great discrepancies [4], the option to extract the upper second right premolar depended on its position and crowding in agreement with Araújo and Caldas [13]. However, space closure in asymmetric extraction presents challenges related to anchorage control. The closure of the post-extractive space and the correction of the midline require an optimal manage of the anchorage; two main situations were identified: anchorage loss of molars during space closure after premolar extraction and anchorage loss in the incisor or premolar region during distal movement of molars [14]. A third anchorage category is skeletal anchorage with miniscrews. The term orthodontic anchorage was first introduced by Edward Angle and can be explained as resistance to unwanted movement [15]. The desire to have complete control over anchorage is no doubt universal among orthodontists [16], in fact, in order to deserve an ideal first class occlusion while conducting the necessary asymmetrical movements, in this case it has been crucial to maintain the proper anchorage control, utilizing lace back, band back, and lingual arch without miniscrews. An alternative treatment could be the use of mini-screws applied mesially to the second premolars at the mucogingival junction, which allow to simultaneously retract the premolars and canines and subsequently the incisors and to center the midline. Systematic reviews and meta-analysis demonstrated that the use of mini screws is strongly recommended in cases of maximum anchorage than other methods or combinations of treatments and it is associated with a shorter duration treatment time [17–19]. Whereas other reviews and meta-analysis showed that miniscrews have an acceptably low failure rate linked to insertion sites [20], to minidesign of screws [21], and to cortical bone thickness [22].

In this case, after premolars extractions, most of the space was used for midline correction, as well coil spring open between teeth #21 and #22, lacebacks between teeth #27 and #23 to distalize the canine and coil spring open between teeth #11and #21, in the lower arch a coil spring closed between teeth #47 and #43 to close post-extractive space. The use of coils as force delivery systems for these kinds of dental movements is well documented in literature as reported in several studies which found them faster in canine retraction compared with elastomeric powerchains with the cost of more canine tipping and rotation and more molar tipping [23, 24]. As showed by the study of Sueri and Turk [25], the use of laceback ligatures proved to be effective for canine distalization. Less canine and molar movement was found for the laceback group, but more controlled movements were obtained for the sagittal, vertical, and transverse planes.

At the beginning of the treatment patient presented skeletal class I, molar class I on the left side and canine class II, molar class I on the right side and canine class III relationship; at the end of the treatment the patient presents skeletal, molar, and canine class I, resolution of crossbite, an improvement of skeletal relationships both in the sagittal and transversal planes. Overall, the straight-wire mechanics associated with coils in this case report achieved a good occlusion.

## 5. Conclusion

To obtain stable results at the end of the treatment, especially while treating midline deviations, it is advisable to pay attention at the correct diagnostic phase and treatment plan, in order to select properly the suitable case. The midline deviations were successfully treated by premolars extractions, coils, and laceback, avoiding the use of miniscrew. The excellent functional treatment outcome was also possible due to the patient compliance. This treatment plan is one of the possible valid approaches without the use of miniscrew, even if those are an effective method to correct the midline deviations as well documented in literature. In case which patient declines orthosurgical alternatives, this less invasive protocol is well accepted and the result we obtained shows the effectiveness of the treatment.

## Figures and Tables

**Figure 1:**
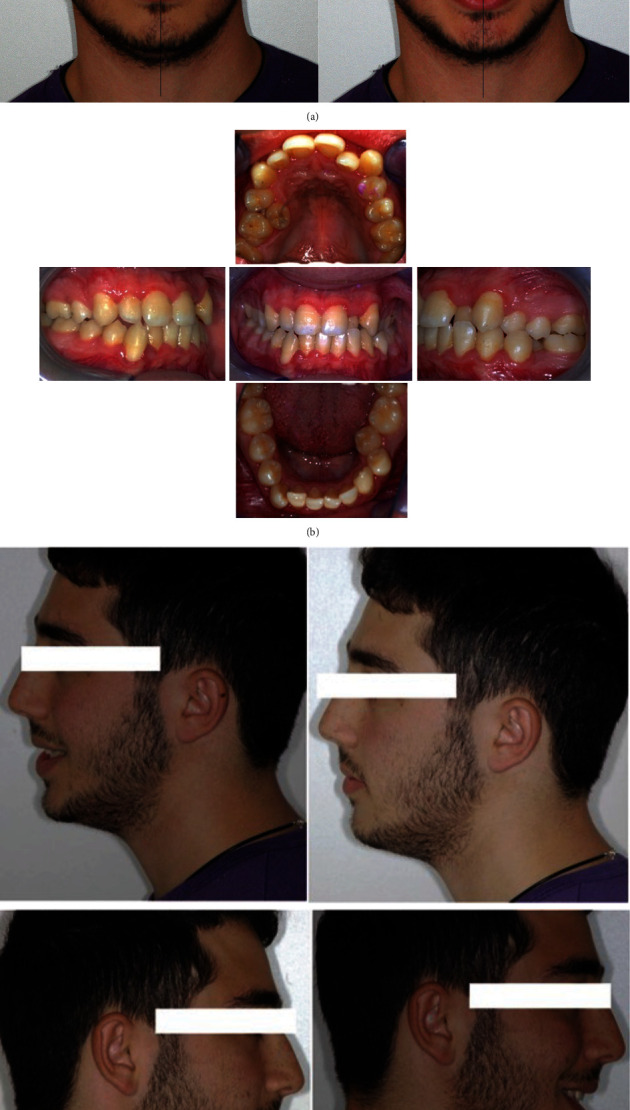
(a) Initial extraoral frontal rest—extraoral frontal smile and initial extraoral left and right profile photographs. (b) Pretreatment intraoral upper and lower occlusal image, right and left lateral photographs. (c) Pretreatment model cast.

**Figure 2:**
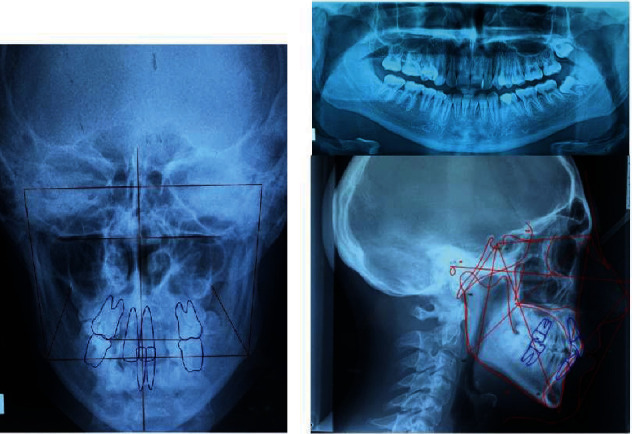
Pretreatment panoramic, antero-posterior, and cephalometric radiographs.

**Figure 3:**
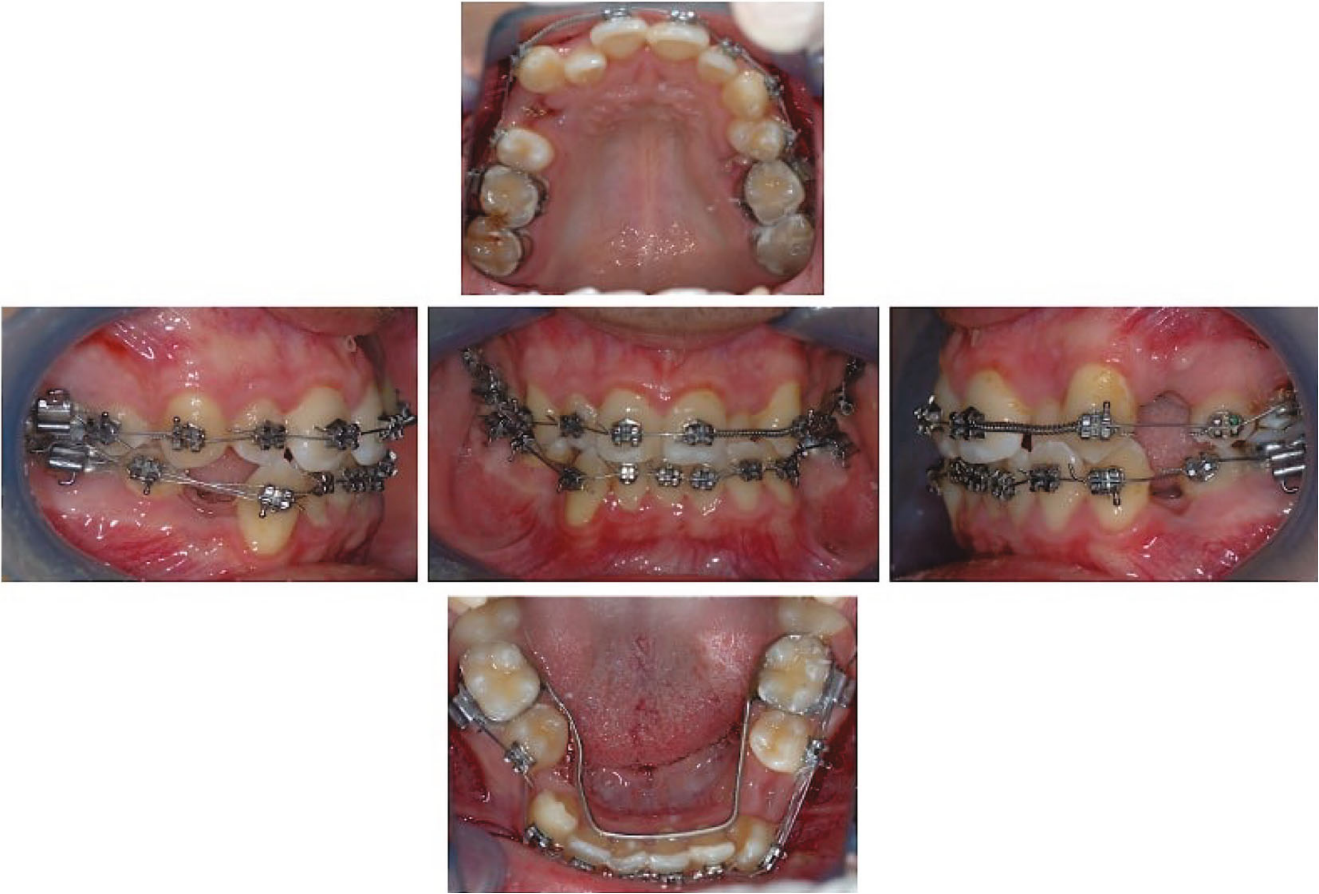
Intraoral views of treatment. Start of leveling and extraction of teeth #14, #25, #34, #44 and lingual arch for anchorage. Lace back between teeth #47 and #43 to distalize the canine, coil spring open between teeth #21 and #23 applied to close the extraction space and to correct the midline deviation. 0.020 × 0.020 NiTi wire upper and lower arch with band back.

**Figure 4:**
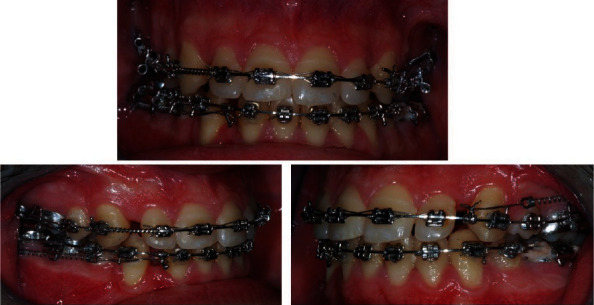
Intraoral views of treatment. Coil spring open between teeth #12 and #13 to correct midline. In lower arch, coil spring closed between teeth #47 and #43, and hook to close post-extractive space. 0.019 × 0.025 stainless steel wire in upper arch with superspee and surgery arch lock in distal position to teeth #22 to close the post-extractive space. 0.016 × 0.022 NiTi wire in lower arch with antispee.

**Figure 5:**
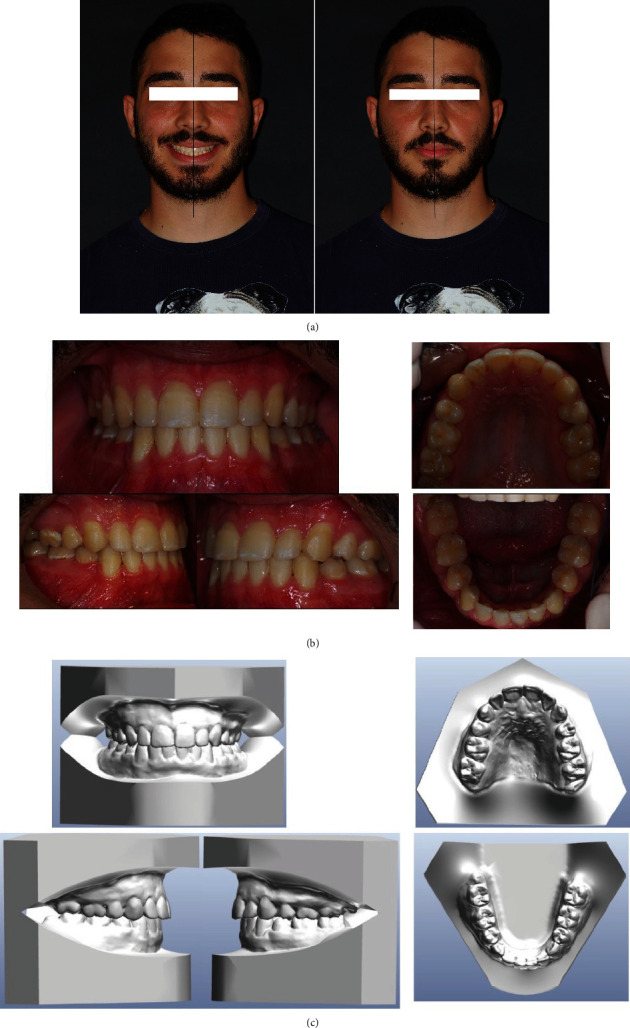
(a) Post-treatment extraoral frontal rest—extraoral frontal smile and extraoral left and right profile photographs. (b) Post-treatment intraoral upper and lower occlusal image, right and left lateral photographs. (c) Final model cast.

**Figure 6:**
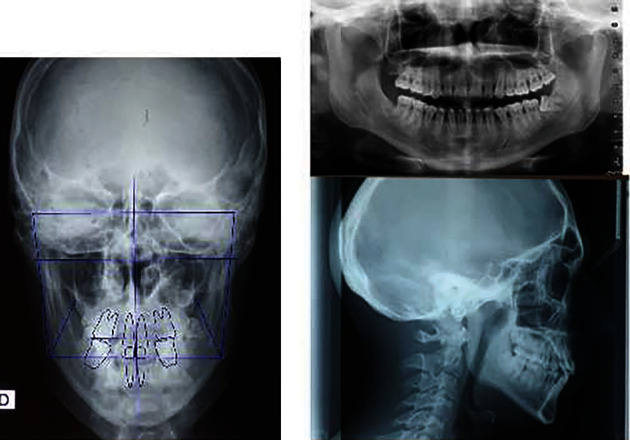
Post-treatment panoramic, antero-posterior, and cephalometric radiographs.

**Figure 7:**
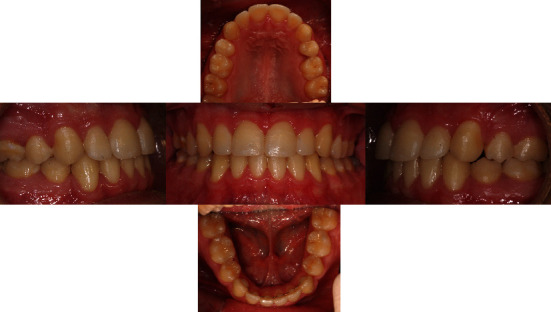
Five-year follow-up.

**Table 1:** Cephalometric analysis (Jarabak) before and after the treatment.

Measurement	Initial	Final	Normal
S^N^A (°)	76.1	76.7	81
S^N^B (°)	73.6	74.6	79
A^N^B (°)	2.5	2.1	2
SN^^^Pog	74.1	74.9	80
S (°)	126.5	127.2	123 ± 5
AR (°)	150.3	150.6	143 ± 6
Go (°)	123.5	122.8	130 ± 7
Upper gonial angle (°)	45.7	46.2	52–55
Lower gonial angle (°)	77.8	76.7	70–75
N^S^Ar^Go^Gn (°)	400.3	400.6	396
Ans-Pns/Go-Me (°)	40.3	40.6	32
SN (mm)	69.9	68.4	71
GoMe (mm)	78.6	78.6	71
S-Ar (mm)	42.3	41.6	32
Ar-Go (mm)	43.1	39.1	44
Dentoalveolar compenent			
UI^SN (°)	100.45	104.67	102 ± 2
LI^GOME (°)	91.7	89.62	90 ± 3
Interincisal angle (°)	127.57	125.09	135
UI^NPog (mm)	8.77	5.69	+2/+4
LI^NPog (mm)	4.74	2.17	−2/+4

**Table 2:** Posterior–anterior cephalometric analysis (Ricketts) before and after the treatment.

Measurement	Initial	Final	Normal
Craniofacial relation			
Postural symmetry (°)	0.6	−0.5	0 ± 2
Deep skeletal structure			
Nasal width (mm)	26.8	26.8	25 ± 2
Nasal height (mm)	75.5	75.5	44.5 ± 3
Maxillary width (mm)	66.1	66.1	61.9 ± 2
Mandibular width (mm)	93.1	93.1	76.1 ± 2
Facial width (mm)	116.8	116.1	116 ± 3
Maxillo-mandibular relationships			
Frontal convexity, left (mm)	15.9	16.6	7.7
Frontal convexity, right (mm)	13.9	16.4	7.7
Maxillo-mandibular midline (mm)	2.4	−2.0	0.7
Skeletal/dental			
Occlusal plane tilt (°)	4.6	−0.1	0 ± 2
Molar to jaw, left (mm)	15.6	21.2	6.3 ± 1.7
Molar to jaw, right (mm)	18.8	18.3	6.3 ± 1.7
Denture to jaw midline (mm)	0.0	−0.3	0 ± 1.5
Dental relationships			
Molar relation, left (mm)	0.5	−1.6	1.5 ± 2
Molar relation, right (mm)	0.4	1.6	1.5 ± 2
Intermolar width, lower (mm)	47.4	44.2	55 ± 2
Intercuspid width, lower (mm)	18.7	21.2	22.7 ± 2
Denture midline discrepancy (mm)	–2.5	0.1	0 ± 1.5
GA—Menton (mm) right side	57.1	57.3	48.7
AG—Menton (mm) left side	55.0	55.5	48.7

## Data Availability

Data supporting this research article are available from the corresponding author or first author on reasonable request.
